# Voluntary and non-remunerated blood donation by 2025? A comparative study of blood donor eligibility in 19 healthcare institutions in Lebanon

**DOI:** 10.1177/13591053251408144

**Published:** 2026-01-23

**Authors:** Samira N. Chatila, Eva-Maria Merz

**Affiliations:** 1 Vrije Universiteit Amsterdam, The Netherlands; 2 University of Minnesota, Minneapolis, MN, USA; 3 Sanquin, Amsterdam, The Netherlands

**Keywords:** blood donation, donor eligibility, voluntary and non-remunerated blood donation (VNRD/VNRBD), health equity, Lebanon

## Abstract

Eligibility criteria in blood donation are essential to protect both donor and recipient health. This study examines variability in donor selection criteria and donor health questionnaires across 19 healthcare institutions in Lebanon amid its transition toward a voluntary and non-remunerated blood donation (VNRD) system. Notable discrepancies were identified across medical, behavioral, demographic, and geographic deferral categories. A comparative analysis against Lebanese Committee for Blood Transfusion guidelines and EU-based standards reveals a lack of centralization and inconsistent adherence to evidence-based practices. Many institutions apply precautionary deferrals not grounded in current science, leading to overly restrictive outcomes. Ethnoracial biases are also evident, with policies that disproportionately exclude migrant groups. These inconsistencies risk confusing donors, reducing trust, and undermining donor retention. The findings highlight the urgent need for standardized, equitable, and medically sound eligibility criteria. This research offers critical insights for policymakers in Lebanon and other countries navigating similar reforms toward sustainable VNRD systems.

## Introduction

The World Health Organization (WHO) and the International Federation of Red Cross and Red Crescent Societies have called for the global elimination of family/replacement and paid blood donation systems by 2025, advocating instead for voluntary and non-remunerated blood donation (VNRD) as the most sustainable and ethical model (WHO, 2017a). A key factor in achieving VNRD is the consistency of donor eligibility policies across healthcare institutions (HIs). While individuals decide whether to donate, their experiences – shaped by recruitment, questionnaires, selection criteria, and deferral outcomes – influence perceptions of eligibility and willingness to donate (Holloway, 2023; Sandner et al., 2021; Thorpe et al., 2024). As this study shows, a donor accepted at one hospital may be permanently deferred at another, creating confusion and undermining the perceived legitimacy of the VNRD system. A transparent, standardized framework can build trust and support sustained donor engagement by addressing the complex interplay between individual motivation and institutional regulation (WHO, 2017a).

While past research has focused on donor compliance, retention, and recruitment (Cutts et al., 2020; Piersma et al., 2017), it has also emphasized how contextual and institutional factors shape donor behavior. Tailored communication strategies and questionnaire design are known to improve engagement (Holloway, 2023; Sandner et al., 2021). Beyond individual factors, institutions play a foundational role: Healy (2006) situates donation within moral economies, highlighting how infrastructures and institutional logics mediate altruism. Gorleer et al. (2020) extend this view, showing how bureaucratic norms and transnational policy frameworks shape participation across Europe. These studies position donation not merely as a biomedical or behavioral act, but as a structurally contingent process shaped by how institutions define and operationalize eligibility.

In this context, Lebanon’s decentralized blood system offers a critical case. Although it records the highest whole blood donation rate in the Eastern Mediterranean (29 per 1000 people), its VNRD rate is among the lowest – just 4.1% (WHO, 2017b). With no central national blood bank, the country relies largely on fragmented, hospital-based services and family or replacement donations (Lebanese Ministry of Public Health [LMOPH] , 2022). To align with WHO goals, Lebanon aims to raise hospital-based VNRD to 30% and Lebanese Red Cross (LRC) center-based VNRD to 60% by 2025 (LMOPH, 2022).

This study examines how Lebanese HIs implement donor selection and deferral policies, focusing on both formal regulations and on-site practices. We analyze 19 institutions – 18 hospitals and the LRC (which oversees 13 centers and is treated as one entity) – to identify structural and contextual influences on donor eligibility.^1^ This comparison is based on two key research questions:

1. How do donor selection criteria and health questionnaires vary among the 19 healthcare institutions in Lebanon, and what does this indicate about their level of centralization?
2. To what extent are variations in these criteria and questionnaires considered to be evidence-based and medically sound?

Lebanon’s evolving VNRD strategy makes this case study especially timely, offering insights relevant to countries undergoing similar reforms. In addition to informing public health and medical policy, this research contributes to health psychology by examining how institutional policies and deferral practices shape donors’ cognitive, emotional, and behavioral responses.

### Centralized and decentralized donor selection criteria

Eligibility criteria are essential to blood donation systems, primarily protecting donor and recipient health by minimizing risks such as transfusion-transmissible infections and adverse donor reactions. They also preserve the integrity of the blood supply and foster public confidence in donation systems. Transparent, evidence-based criteria help build trust in blood collection agencies (BCAs), which is critical for sustaining VNRD. Research shows that during times of uncertainty, such as the COVID-19 pandemic, trust in BCAs enables continued donor participation and supports broader donor engagement (Haw et al., 2022).

Blood systems operate under centralized or decentralized models, each influencing VNRD in distinct ways. In centralized systems, such as those in Iran or Kuwait, a single national agency oversees blood collection and distribution, setting nationwide standards for eligibility, operations, and quality control. In decentralized systems, such as those in Germany or the United States, multiple independent blood centers operate regionally with greater policy autonomy, while still adhering to overarching national safety regulations. These structural differences affect donor recruitment strategies, data management, and standardization of care (Gorleer et al., 2020). Harmonized donor eligibility enhances screening consistency, reinforces trust, and encourages sustained donor participation (Sandner et al., 2021).

### Donor deferral rationales: Precautionary measures and evidence-based practices

Balancing precaution and evidence is critical to ensuring donor and recipient safety in blood systems (Brailsford et al., 2015). Historically, deferral policies reflected a zero-risk approach in response to infections like HIV and HCV, but there is a growing shift toward risk-based models that integrate science, ethics, and social values (Menitove et al., 2014). The donation process includes registration, eligibility screening, a health assessment, donation, and a post-donation rest period, with deferrals possible at any stage. Deferrals generally fall into two categories: precautionary deferrals, applied amid scientific uncertainty or emerging risks, and evidence-based deferrals, grounded in medical, behavioral, or geographic criteria.

Some deferral policies, particularly those addressing uncertain or emerging risks, are precautionary and may impose stricter criteria that reduce the VNRD donor pool (De Kort et al., 2016). However, not all contested deferrals fall into this category. For instance, long-standing eligibility restrictions for Men who have Sex with Men (MSM) were initially based on HIV risk and the scientific evidence available at the time (Lewin et al., 2024). While rooted in legitimate risk assessments, these policies often differed in duration and framing from those applied to heterosexuals with new or multiple partners, raising concerns about consistency and equity (Galarneau, 2010). Advances in HIV testing have led many jurisdictions to revise MSM criteria in line with current evidence and risk-based frameworks (Fisher and Schonfeld, 2010). Similar inconsistencies appear in deferrals for conditions like epilepsy, where regional variation underscores the ongoing challenge of maintaining fair, evidence-aligned screening (Kellens et al., 2018).

In contrast, evidence-based deferrals rely on scientific justification, typically categorized into medical, behavioral, and geographic criteria (Galarneau, 2010; Mikkelsen et al., 2021). *Medical deferrals*, the most reported reasons for deferral, aim to protect donor and recipient health in cases involving underlying conditions, treatments, medications, or risk of transfusion-transmitted complications.^2^ *Behavioral deferrals*, constituting the second most frequently cited category of donor deferrals, relate to sexual risk behaviors and lifestyle factors that may increase infection risk (Galarneau, 2010).^3^ *Geographic deferrals* concern a donor’s place of birth, travel history and behavior, or residence in areas with prevalent infectious diseases (Galarneau, 2010).

While this typology supports eligibility assessments, it may obscure structural and contextual factors – such as socioeconomic status, healthcare access, and HI-specific practices – that shape deferral decisions and contribute to disparities across HIs. Understanding how institutions apply or diverge from these criteria is critical, particularly where precautionary deferrals result in controversial policies. For example, epilepsy-related deferrals vary globally: some countries impose permanent bans, others allow donation after seizure-free or medication-free intervals, and some accept donors without restriction (Kellens et al., 2018). These inconsistencies are often driven by expert opinion, legal norms, or institutional discretion, rather than scientific evidence. The absence of a consistent, evidence-based rationale raises concerns about fairness, transparency, and the exclusion of potential VNRD donors despite medical advancements (Fisher and Schonfeld, 2010; Kellens et al., 2018).

### Impact of donor eligibility on donor behavior

Deferrals, whether evidence-based or not, disrupt donation routines and often deter return due to personal, logistical, or psychological barriers (Hillgrove et al., 2012). Less experienced donors are particularly affected (Spekman et al., 2019), and many experience negative emotional responses, including anger, frustration, and feelings of rejection (Gemelli et al., 2021), which further reduce the likelihood of future donation. Clement et al. (2021) found that the impact of temporary deferrals varies with donor experience and deferral frequency: while experienced donors adapt over time, each deferral decreases the probability of returning. These findings highlight the need for targeted interventions to re-engage deferred donors (particularly early-stage ones) and to avoid discouraging new donors.

Fear, mistrust, and perceived inconvenience remain persistent deterrents to VNRD, often taking the form of needle anxiety, concerns about adverse reactions, low self-efficacy, or mistrust in healthcare systems (Bednall and Bove, 2011; Graf et al., 2024). These psychological and informational obstacles call for targeted interventions tailored to donor concerns and motivations. Simultaneously, institutional barriers – such as decentralized governance, inconsistent eligibility criteria, and fragmented VNRD policy implementation – undermine accessibility and continuity (WHO, 2012). Addressing both individual and systemic levels is critical to expanding the VNRD donor base. Moreover, donor return is strongly influenced by the donation experience itself, particularly interactions during the health questionnaire, which shape satisfaction and trust (Holloway, 2023; Sandner et al., 2021).

### Lebanese blood donation/transfusion context

Lebanon, with a population of 6.7 million, hosts one of the highest per capita refugee populations globally. Its predominantly private healthcare system has expanded under liberal economic policies (Haddad et al., 2017). Unlike countries with centralized national blood banks, Lebanon’s decentralized blood supply system involves private and public hospitals, the Lebanese Red Cross (LRC), and various NGOs (LMOPH, 2022).^4^ These actors manage donor recruitment, blood collection, testing, and transfusion services.^5^ The system includes LRC-run blood centers and independent hospital-based blood banks, which follow Lebanese, American, or French transfusion guidelines (Haddad et al., 2017).^6^

Annual blood demand ranges from 100,000 to 150,000 units (LRC, 2020), though these figures do not reflect the impact of compounding crises (Supplemental Figure 1) which have severely strained the healthcare system (Jawad et al., 2023). Electricity and fuel disruptions compromise blood storage and reduce availability, increasing dependence on family/replacement donors over voluntary ones. Economic barriers further discourage donation, as donors often bear transport costs, wage losses, and other out-of-pocket expenses (Jawad et al., 2023).

To address this fragmentation, the Lebanese Committee for Blood Transfusion (LCBT), under the LMOPH, issued non-binding guidelines in 2015 and 2018 on donor selection criteria (DSC) and a standardized 51-item donor health questionnaire (DHQ; LCBT, 2018; Lebanese Committee of Blood Transfusion [LCBT], 2015). However, adherence remains inconsistent, particularly across the private institutions. In 2022, LMOPH launched a new 4-year national strategy (2022–2025) focused on strengthening service capacity, regulatory oversight, hemovigilance, and voluntary donor recruitment (LMOPH, 2022). As part of this initiative, stakeholders were mapped, including LMOPH, LCBT, hospitals, the LRC, donor recruitment organizations (DROs), and advocacy NGOs.^7^ Countries like India partially centralized and coordinated its fragmented blood services via national and state councils, raising VNRD from 54.4% to 83.1% over 5 years (Marwaha, 2015).

## Methodology

### Research approach: Multi-sited ethnography

This study is part of a broader ethnographic project (June–September 2022) examining blood donation practices in Lebanon, in line with the 2022–2025 national strategy to transition toward a VNRD system. Using a multi-sited ethnographic design, it focuses on third-party non-governmental DROs that either refer donors to healthcare institutions (HIs) or co-organize blood drives with them. Drawing on observation, interviews, and document collection (Nippert-Eng, 2015), the study explores donor recruitment and selection practices across institutional contexts.

### Data collection

Fieldwork included ethnographic observations, semi-structured interviews, and the collection of internal and public documents from both DROs and HIs (Supplemental Table 1). Two key document sets underpin the analysis: DSC collected from 18 HIs, and DHQ collected from 9 HIs.

#### Call center observations

DRO-run call centers mediate between patients’ families and potential donors. Observations at these sites revealed how DRO staff inferred hospital-specific DSCs based on follow-ups with deferred donors. These criteria – compiled by DROs rather than the HIs themselves – reflect de facto eligibility standards in practice, including exclusions based on medication use, medical conditions, travel history, and ethnoracial classification.

#### Blood drive observations

Observations of blood drives, typically co-organized with HIs, enabled collection of DHQs. At each event, HI staff distributed donor screening forms, which varied across institutions. These variations in form structure, terminology, and emphasis revealed inconsistencies between DHQ content and inferred DSCs. Supplemental Table 2 lists which HIs provided each document type (de-identified by pseudonym).

### Study sample

The 19 HIs studied represent Lebanon’s largely private healthcare sector, delivering most primary and secondary care (Haddad et al., 2019; Jawad et al., 2023). The sample includes hospitals and NGOs involved in transfusion and recruitment across Beirut, Mount Lebanon, Kesserwan-Jbeil, and the South. Institutional affiliations span international agencies, religious organizations (e.g. Lebanese Maronite Order, Sharia-based bodies), and academic hospitals. These affiliations shape distinct ethical, cultural, and medical approaches to blood donation (Supplemental Table 3).

### Data analysis

We analyzed 9 DHQs and 18 DSCs using comparative, thematic, and content analysis. Thematic analysis followed Braun and Clarke’s (2006) inductive coding to identify institutional logics and patterns. Content analysis, based on Hsieh and Shannon’s (2005) directed approach, used Galarneau’s (2010) typology of deferrals – behavioral, medical, and geographic – with an added category for demographic deferrals (e.g. gender, age, weight, childbearing history).

To assess the degree of centralization, we referenced non-binding guidelines from the LCBT (2015, 2018), including a national DSC recommendation and a standardized 51-item DHQ. While not enforced, these documents are widely adopted or adapted by HIs and serve as benchmarks for evaluating consistency. To evaluate the medical soundness of deferral variations, we drew on the TRANSPOSE (TRANSfusion and transplantation PrOtection and SElection of donors) framework (Mikkelsen et al., 2021; Sandner et al., 2021), developed by an EU-funded consortium of 25 partners across 15 countries. Given that Lebanese professionals often align with European standards (Haddad et al., 2019), this framework provides a relevant and robust point of reference.

### Ethical considerations

This study, part of the research project entitled “Blood Donation and Donor Behavior Beyond the West: A Case Study of Lebanon,” was reviewed by the Research Ethics Review Committee (RERC) of the Faculty of Social Sciences at the Vrije Universiteit Amsterdam, and it was determined to be exempt from further ethical review. The ethics check was conducted under reference number 2022-4-28-613.

## Results

This section presents findings from our analysis of 9 DHQs and 18 DSCs across 19 Lebanese HIs. We begin with a structural analysis of DHQs, followed by a thematic and content analysis of DSCs, with references to DHQs where relevant. Since DSCs are shaped primarily by DROs rather than official HI policies, they are excluded from the structural analysis.

### Structure of DHQs

In the DHQs we analyzed, there are four key sections, although their order can vary (see Supplemental Table 4). Typically, the first section is designed to gather personal information from the donor, such as their name, age, and contact details. The second section, filled out by the HI staff, focuses on the physical examination of the donor, documenting any relevant findings. The third section is composed of a series of questions that form the core of the DHQ, inquiring into the donor’s health history and other pertinent information. In the final section the donor signs a declaration statement, affirming the truthfulness and accuracy of their provided information.

#### Donor information

The first section of the DHQ gathers personal information, with content varying across institutions. Most HIs (8 of 9) requested the donor’s full name (typically including first, father’s, and family names), with two also asking for the mother’s name, as well as contact details like address and phone number. Seven HIs requested date of birth and nationality or citizenship, while six collected sex/gender data, limited to “male” or “female.” All DHQs required the full name of the intended recipient and the donor–recipient relationship. Four HIs inquired about the voluntariness of the donation, and HI6 included this reference in the declaration statement (Section 4). Supplemental Table 5 summarizes these elements.

#### Physical examination assessment

The second section of the DHQ, completed by HI staff, covers the physical examination of the donor (Supplemental Table 6). Weight, temperature, blood pressure, pulse, and hemoglobin were requested by 8 of 9 HIs, whereas height and blood type appeared in only 2 of 9 HIs. General appearance was checked by 6 of 9 HIs, and skin lesions by 7 of 9 HIs. Only 6 of 9 HIs asked about needle marks on the arms. Most HIs (7 of 9 HIs) recorded the names of the staff who conducted the physical exam or collected the blood, indicating a fairly uniform practice of accountability.

#### Donor history

The third and most substantive section of the DHQ covers donor health history. Most HIs provide the form in both Arabic and English to accommodate a bilingual donor base. Supplemental Table 7 summarizes core features across DHQs, including language, total number of questions, and deviations from the standard LCBT version. Question counts ranged from 21 to 53, reflecting variation in screening depth.

Private HIs have broad discretion to modify the LCBT’s DHQ, resulting in extensive customizations that reflect institutional priorities, risk assessments, and evolving medical practices. Across HIs 2, 4, 6, 10, and 17, commonly retained items – such as those on medications, pre-donation meals, pregnancy, and dental procedures – were often revised in terms of timeframe or specificity. Conversely, questions related to sexual health risks (e.g. contact with individuals with hepatitis or histories of injection drug use), malaria-endemic travel, and informed consent (e.g. HIV transmission) were frequently removed, simplified, or merged into broader categories.

In addition to deletions and modifications, HIs 2, 4–5, 10 and 17 introduced new items addressing COVID-19 exposure, chronic conditions, and specific infectious diseases such as babesiosis and Chagas. Other additions included extended travel and residence histories (e.g. in Europe and Africa), personal health factors (e.g. hypertension management, traditional treatments like acupuncture), previous donation experiences, and lifestyle considerations such as alcohol use and fear of needles. These revisions also involved combining questions, adjusting deferral periods, or eliminating items entirely.

#### Statement of declaration

Analysis of DHQ declaration statements reveals a common structure across several institutions (LCBT, HIs 2, 11–13, 19). These typically require donors to certify the accuracy of their medical history, acknowledge receipt of educational materials on high-risk behaviors, affirm they are not at increased risk for transmitting infectious diseases, and consent to blood withdrawal and testing. A consistent clause also instructs donors to remain on-site for 10 minutes post-donation, highlighting the consequences of leaving early. This uniformity suggests a standardized approach to promoting donor responsibility, safety, and legal compliance. However, other HIs introduce distinct emphases. HI4, for instance, underscores the ethical responsibility of honesty in its streamlined declaration:

I, the undersigned, certify that the information I have provided is correct, accurate, and complete to the best of my knowledge. I understand that answering the above questions clearly and honestly contributes to my safety and to that of the person for whom I am donating blood. (HI4).

This contrasts with declarations from HIs 2, 11–13, 17, and 19, which include questions such as “Are you giving blood because you want to be tested for HIV/AIDS virus?” – absent from the DHQs of HIs 4, 6, and 10 – suggesting varying assumptions about donor intent.

HI6’s declaration uniquely emphasizes voluntariness, non-remuneration, and legal transfer of ownership:

I, the undersigned, declare that the above information is true and I take full responsibility in case it proved otherwise. I understand that blood donation is a totally voluntary act and that no inducement or remuneration has been offered. Donation of blood components is a medical procedure and by donating voluntarily, I accept the risks associated with it. I have also understood that the donated blood will be the property of the [HI] and that in case the blood was not given to the designated patient, I will not have the right to take back any blood units. (HI 6).

This language directly addresses potential misconceptions around ownership and retrieval of donated blood. Supplemental Table 8 summarizes the variations in declaration statements across all HIs.

### Donor deferral categories

We categorized deferral reasons into four main types: medical (health conditions), behavioral (lifestyle), geographic (travel or residence), and demographic (e.g. age, gender). Our comparative analysis assessed deferral outcomes across institutions, ranging from Immediate Donation (ID) to Permanent Deferral (PD) and temporary deferrals (in days, weeks, months, or years). To capture this variation, we developed the Deferral Period Range Interval (DPRI) – the span between the shortest and longest deferral periods recorded for each criterion. This framework enabled benchmarking of deferral practices across the 19 HIs against LCBT recommendations and TRANSPOSE’s evidence-based standards. Using the DPRI, we identified inconsistencies within DHQs relative to LCBT norms and evaluated the medical validity of DSCs through comparison with TRANSPOSE guidelines.

#### Medical deferrals

Medical reasons for deferring donors primarily entailed recent vaccinations as well as medical conditions and medications that the potential donor was consuming at the time of presenting for donation.

##### Vaccinations

Deferral periods following COVID-19 vaccination varied widely across HIs, with deferrals ranging from immediate donation (no deferral) to deferral for 60 days, depending on the vaccine type and dose (Supplemental Table 9). Notably, deferral times tended to decrease after subsequent doses.

For non-COVID vaccines, deferral practices also varied widely (Supplemental Table 10). While LCBT guidelines permit immediate donation for HPV and influenza vaccines, some HIs imposed deferrals of up to 3 months for HPV and extended influenza vaccine deferrals beyond the LCBT’s 28-day recommendation. The most striking variation was observed with the DPT/Tdap vaccine, where the LCBT allowed immediate donation, but some HIs imposed deferrals of up to 12 months, indicating a more conservative approach.

##### Medications and medical conditions

The DHQ items reflected a broad approach to assessing donor suitability (Supplemental Table 11), covering both past and current medication use over varying timeframes. The questionnaire paid attention to treatments for both chronic and acute conditions, including infectious diseases like hepatitis and syphilis. Medications that might alter blood properties, such as aspirin and clotting factor concentrates, and hormone treatments, were also scrutinized. While the DHQ is comprehensive, it cannot cover every possible medication and medical condition. Thus, decision-making often depends on DSCs, particularly regarding medication-related deferral periods (Supplemental Table 12), which vary based on condition severity and treatment type.

For example, the deferral period for acne treatment ranged from 1 month to 3 years, indicating a cautious approach due to the condition’s severity or medication side effects (Supplemental Table 12). Still, LCBT allowed donors with acne to donate blood immediately under the condition that the venipuncture site is unaffected, and the patient is not treated with Roaccutane. For other conditions like allergies, asthma, cholesterol issues, diabetes, epilepsy, hypertension, mental health disorders (including ADHD, Bipolar disorder, Depression, Schizophrenia), thyroid problems, and high triglycerides, the deferral range was also extensive, from immediate donation to permanent deferral.

#### Behavioral deferrals

The behavioral reasons entailed variation in gender related blood and/or platelet donation intervals, lifestyle choices, and high-risk sexual behavior.

##### Donation intervals

Donation intervals varied by donor gender and the type of previous donation (Supplemental Table 13). On average, whole blood donations were spaced 2.3 months apart for males and 2.9 months for females – both exceeding the LCBT’s 2-month guideline. Platelet donation intervals showed no gender difference, averaging 14.5 days, well above the LCBT’s 2-day minimum (applicable only when red blood cells are fully returned; otherwise, an 8-week deferral applies). The average interval between a platelet and subsequent blood donation was nearly 17 days for both genders, again exceeding the 2-day recommendation under the same condition. These averages reflect data from hospitals that accepted female donors, as some HIs restricted women’s eligibility to donate whole blood or components.

Out of 18 HIs, 8 imposed specific restrictions on female donors. Several hospitals (HIs 3, 10–13, 16) disqualified women who are or were married and have children from donating entirely or from donating specific components such as platelets or plasma. In the case of H15, women were barred from donating altogether. Additional limitations included time-based restrictions (e.g. H14 only accepting female donors before 3 p.m.) and broad exclusions from apheresis donations (platelets and plasma; HIs 11–12, 14, 16).

##### Lifestyle

Various lifestyle-related deferrals, including hair removal and *Houjama*,^8^ show considerable variation across HIs (Supplemental Table 14). For *Houjama*, the LCBT set a 1-year deferral period, which was followed by 5 HIs (HIs 5, 8, 11, 17–18). However, 6 HIs (HIs 2, 6–7, 9, 14–15) implemented shorter deferral periods ranging from 2 to 6 months.

For hair removal, particularly laser or electrical epilation, deferral outcomes were almost evenly split: 6 HIs (HIs 2–4, 9, 12–13) allowed immediate donation, while 7 (HIs 5–7, 10, 16–18) imposed temporary deferrals ranging from 1 month to 1 year. Notably, LCBT’s DSC did not include any restrictions on hair removal.

As for quasi-medical consumables, there was no explicit LCBT guidance on the consumption of protein shakes, creatinine, fat burners, and weight loss medications, yet some HIs introduced their own deferral criteria for these substances.

##### High risk sexual behavior

Our DSC dataset did not include deferral outcomes for high-risk sexual behaviors. However, a comparative content analysis of DHQ items provided a proxy means to assess how these behaviors are evaluated across institutions. This analysis revealed variation between HIs, the LCBT, and TRANSPOSE (Supplemental Table 15). TRANSPOSE’s questions were concise and time-bound, focusing on high-risk behaviors within the past 6 months. In contrast, the LCBT and affiliated HIs posed questions that were either overly broad (e.g. sexual contact with people with hemophilia) or overly specific (e.g. sexual contact with prostitutes), potentially leading to unnecessary exclusions.

The specificity of some questions, particularly those related to geographical locations or long-past behaviors, seems outdated and not reflective of current medical evidence. For instance, questions focusing on sexual contact with individuals who have lived in Africa (HIs 2, 6, 17) are notably precise and could be perceived as unnecessarily stigmatizing. HI2’s question, “Have you ever had sexual contact with anyone who was born or lived in Africa?” exemplifies this. The undefined timeframe, and the inclusion of “or *born* in Africa” conflate birthplace with elevated risk, regardless of actual behavior, potentially reinforcing harmful stereotypes. Broad questions like HI6′s “have you *ever*” fail to account for the temporality of risk and should be revised to reflect current evidence and avoid discriminatory assumptions.

#### Demographic deferrals

##### Upper age limit

The LCBT DSC set the upper age limit for male and female blood donors at 60 years. However, we found variation in this limit across the 18 HIs analyzed, ranging from 50 to 65 years for both males and females. Notably, gender-specific differences in age limits were observed in 6 of these institutions (HIs 3, 4, 5, 6, 7, 8), whereby males and females had differing upper age limits within the same HI.

##### Minimum donation weight

In terms of weight, while the LCBT guidelines specify a uniform minimum of 50 kg for donor eligibility regardless of gender, the actual practice varied (Supplemental Table 16). The minimum weight requirement for donation among the surveyed institutions ranged from 50 to 65 kg for males and 50 to −70 kg for females. This variation in weight criteria, exhibiting gender differences, was evident in 13 of the 18 HIs.

##### Gender-specific deferrals

Among the 18 HIs, 8 imposed restrictions on female donors. For example, HI5 restricted donations during menstruation, disregarding evidence against menstrual blood loss impacting safety. The policy at 6 institutions (HIs 3, 10–13, 16) barring women with a pregnancy history seems outdated, as recent studies (Edgren et al., 2019) find no increased mortality risk in transfusions from previously pregnant donors. Our findings also revealed that at HI 15, there is a complete ban on female blood donors, and at HIs 12 and 14, there are specific restrictions on women’s plasma or platelet donations.

#### Geographic deferrals

Finally, the geographic deferrals entailed demographic factors as well as history of travel and residence.

##### Travel restrictions

The LCBT has identified travel destinations where blood donation is subject to restrictions due to certain disease risks, namely Malaria, West Nile Virus, Chikungunya, Chagas, and Dengue. Supplemental Table 17 shows that some HIs have applied their DSC to a broader range of geographic regions than those outlined by the LCBT’s DSC. Additionally, there are notable inconsistencies in the deferral periods assigned to countries with identical risk factors. In some instances, these align with LCBT’s conditional deferrals, like with Malaria, yet contradict in others, notably with Chagas disease. For example, Brazil, Hungary, and Mexico, all Chagas-endemic countries, have divergent deferral periods ranging from immediate donation to permanent deferral, despite the LCBT DSC advocating for a uniform outcome of permanent deferral for Chagas-risk regions. Moreover, certain geographic regions not listed by LCBT as having specific disease risks, such as Algeria, Egypt, Syria, and “Europe,” are still arbitrarily assigned a range of deferral periods by HIs, from immediate donation to permanent deferral.

The diverse criteria in assessing travel-related risks become more apparent when examining the travel history components of the DHQs used by various entities, including LCBT, TRANSPOSE, and HIs, as shown in Supplemental Table 18. For instance, TRANSPOSE, asks basic questions about travel outside the country in the past year, with a specific emphasis on stays in the UK, due to concerns about variant Creutzfeldt-Jakob disease (vCJD). The LCBT and HIs (HIs 11–13, 19) extend this timeframe to the past 3 years, including queries about stays in the UK, blood transfusions in Europe, and visits to malaria-endemic countries. This broader scope reflects heightened concerns about blood safety and prevalent diseases. Other HIs further expand their inquiries to include prolonged stays in Europe, travel or residence in African countries, and sexual contact with African residents, once again indicating a possibly outdated and stigmatizing perception of disease prevalence in Africa. The time frames covered in these questions vary widely, with some focusing on specific historical periods, notably the 1980–1996 period in the UK, reflecting a particular concern linked to vCJD. However, some questions, especially those targeting Africa and long stays in Europe, appear outdated and overly cautious. This is especially evident in the use of open-ended queries like “have you *ever*,” which lack a specific timeframe.

##### Ethnoracial deferrals

The DSC of LCBT does not differentiate based on citizenship or nationality. In stark contrast, our study revealed that the DSC of various HIs imposed definitive outcomes—either immediate donation eligibility or permanent deferral—directly targeting nationalities that are predominant among migrant domestic workers in Lebanon. This demographic, primarily from Asian and African countries, including the Philippines, Ethiopia, Bangladesh, Sri Lanka, Nigeria, Egypt, Ghana, and Kenya, occupies a significant yet vulnerable tier in Lebanon’s socio-economic fabric (Kassamali, 2021; MSF, 2023).

Our study uncovered disparities in the HIs’ DSC. Six HIs (HIs 1, 4–5, 10, 13, 17) disallow Egyptian nationals from donating blood, and four HIs (HIs 4, 5, 8, 17) prohibit Iraqi nationals. Most notably, HI 17 implements a blanket permanent deferral on broad, non-specific groups such as “Africans, Chinese, Ethiopians, Bangladeshis, and Asians,” and HI 18 permanently defers Filipino nationals.

## Discussion and conclusions

Lebanon’s decentralized blood donation system, with its fragmented policies and institutional autonomy, exemplifies how healthcare governance shapes public health outcomes. Our findings suggest a wide variety of variations in DSC and DHQ, which can create inconsistencies in who is eligible to donate blood across different HIs, thereby potentially creating structural inconsistencies that are likely to complicate the transition into a VNRD system.

The variation in DSC and DHQ across institutions reflects broader dynamics of medical authority and institutional discretion in Lebanon. Our findings indicate that this hypervariation fosters uncertainty and inequity in donor selection. Institutions often make discretionary deferral decisions based on demographic, medical, behavioral, or geographic factors – frequently without clear medical justification. The exclusion of certain groups, such as migrant workers, appears arbitrary and raises ethical concerns about racialized and class-based barriers to participation in the blood donation system. These findings align with Galarneau’s (2010) argument that donor deferral decisions are not purely medical but are shaped by sociocultural norms, institutional practices, and historical precedents.

Moreover, these inconsistencies create confusion among potential donors, affecting their willingness to donate (Davison et al., 2020). The lack of a centralized, transparent system fosters distrust, as individuals may experience different eligibility outcomes at different institutions. When donors experience inconsistent eligibility criteria, they may perceive the system as arbitrary – undermining their willingness to return and eroding trust in the healthcare system, a key predictor of donation behavior (Graf et al., 2024). This can be particularly damaging to donor retention, as previous research (Clement et al., 2021) suggests that negative donation experiences reduce the likelihood of repeat donation, particularly among first-time donors. As Lebanon moves toward a VNRD model, ensuring standardization in DSC and eligibility screening is crucial for maintaining public confidence and expanding the donor pool.

### Tensions between precautionary measures and evidence-based deferrals

Our study highlights the problematic overlap between medical conservatism and precautionary deferral practices. The wide variation in deferral periods for COVID-19 vaccination, travel to endemic regions, and common conditions such as acne, hypertension, and mental health disorders suggests that many Lebanese institutions apply overly restrictive criteria that exceed evidence-based guidelines. Although precautionary deferrals are intended to address scientific uncertainty (De Kort et al., 2016), excessive caution risks unnecessarily narrowing the donor pool and disproportionately excluding certain groups without robust medical justification.

A striking example of this is the variation in travel-related deferrals, with some HIs imposing indefinite or excessively long deferral periods for donors from (or who have visited) specific regions. Although intended to reduce transfusion-transmitted infection risks, these deferrals often rely on outdated or overly broad epidemiological assumptions. The continued blanket exclusions of entire populations, such as “Africans” or “Asians,” illustrate how institutionalized risk assessments can perpetuate racialized exclusions, echoing concerns about discriminatory donor policies (Fisher and Schonfeld, 2010). Such practices not only shrink the donor pool but also stigmatize and alienate marginalized communities.

On the other hand, certain HIs adopt more lenient deferral periods for practices like *Houjama*, a procedure with cultural and religious significance. This discrepancy suggests that some deferral policies may be shaped by social and cultural considerations rather than purely medical evidence. While cultural responsiveness is important in healthcare, deferral decisions must remain anchored in epidemiological evidence to ensure both donor inclusivity and transfusion safety.

### Structural barriers to VNRD and the role of donor experience in predicting donor behavior

The transition to a VNRD model requires more than standardized eligibility criteria; it demands confronting the institutional discretion and variable risk assessments that shape donor eligibility. Current inconsistencies in DSC and DHQ mean a donor accepted at one institution may be temporarily or permanently deferred at another, thus creating confusion, frustration, and disengagement. While this study does not capture donor perspectives directly, prior research shows that deferrals, let alone inconsistent ones, can negatively impact donor retention (Hillgrove et al., 2012; Spekman et al., 2019), underscoring the importance of standardization for sustaining a VNRD model.

Despite shared public health objectives, hospitals apply widely divergent risk thresholds. Some follow LCBT guidelines closely, while others impose stricter deferrals without strong medical justification. Conditions like hypertension, mental health disorders, and COVID-19 vaccination are met with vastly different deferral periods, despite limited evidence supporting such variability. Similarly, travel-related deferrals range from temporary to permanent, often reflecting outdated assumptions rather than current evidence on transfusion-transmitted infections.

These inconsistencies create systemic unpredictability, eroding donor trust and undermining the integrity of the donation process (Ferguson et al., 2024). While institutional autonomy allows for context-specific assessments, the lack of binding national standards fosters inequities that may potentially weaken both donor retention and public confidence. Overcoming these structural barriers requires regulatory reform and harmonization of risk assessment frameworks to ensure eligibility decisions are evidence-based, consistent, and equitable.

Compounding this issue, requiring donors to name a recipient on the DHQ reinforces a model of directed and replacement donation thereby contradicting the core principles of VNRD. This practice prioritizes short-term need over long-term sustainability, further obstructing the shift toward a voluntary system. Advancing VNRD will require systemic reform: greater transparency in deferral policies, the removal of unjustified eligibility barriers, and improved donor education to build trust and long-term engagement.

### Implications for healthcare institutions

Strengthening Lebanon’s blood donation system and advancing the shift to a VNRD model requires addressing key structural and procedural gaps. Standardizing DSC and DHQ is essential for ensuring fairness, transparency, and consistency across institutions. The current decentralized system fosters disparities in deferral decisions, leaving potential donors confused and discouraged. Binding national regulations enforced through the LMOPH and the LCBT would reduce institutional variation and create a more predictable, trustworthy donation process.

Deferral policies must also be grounded in up-to-date medical and epidemiological evidence, not excessive precaution. Overly restrictive or outdated criteria unnecessarily shrink the donor pool, undermining both supply and public trust. Institutionalizing regular policy reviews can ensure deferral guidelines remain aligned with global best practices and evolving evidence.

Equally urgent is the need to eliminate discriminatory deferral practices that disproportionately exclude specific nationalities and racial groups. This study’s findings suggest that ethnoracial exclusions lacking medical justification not only violate ethical healthcare standards but also reinforce systemic inequities in donor recruitment. Selection criteria should prioritize individualized risk assessments over broad demographic generalizations.

Donor experience also plays a pivotal role in shaping long-term engagement. Clear communication with deferred donors, minimizing unnecessary exclusions, and offering pathways for re-engagement are essential to improving retention. As frontline actors, physicians and healthcare workers are uniquely positioned to build trust and foster inclusive donation environments.

Finally, public education is critical to dispelling misconceptions about blood donation, especially regarding eligibility. Raising awareness and improving understanding of VNRD can help expand the donor pool, promoting a culture of voluntary donation grounded in medical integrity and social equity.

### Future research directions

Further research is needed to explore the long-term impacts of donor deferral practices on retention rates and public perceptions of blood donation. Additionally, comparative studies between centralized and decentralized blood donation systems could provide valuable insights into best practices for promoting VNRD. While this study focuses on policy and institutional variations, future research should incorporate qualitative data on donor experiences to further elucidate the lived consequences of these disparities in selection and deferral practices. This study contributes a systematic analysis of institutional DSC and DHQ, providing a foundational policy critique. Future work should build upon this foundation by directly engaging with donor narratives to explore how these eligibility inconsistencies impact donor trust, willingness to return, and perceptions of HIs.

## Supplemental Material

sj-docx-1-hpq-10.1177_13591053251408144 – Supplemental material for Voluntary and non-remunerated blood donation by 2025? A comparative study of blood donor eligibility in 19 healthcare institutions in Lebanon Supplemental material, sj-docx-1-hpq-10.1177_13591053251408144 for Voluntary and non-remunerated blood donation by 2025? A comparative study of blood donor eligibility in 19 healthcare institutions in Lebanon by Samira N. Chatila and Eva-Maria Merz in Journal of Health Psychology

## Notes

1. In Lebanon, hospitals and other HIs handle blood collection, storage, and transfusion but rarely recruit donors. Donor organizations focus on outreach and blood drive coordination, typically without collecting or storing blood themselves. The Lebanese Red Cross is an exception, managing recruitment, collection, storage, and distribution through its own blood banks.

2. The most common medical deferral reasons in Global South regions include low hemoglobin, infections (e.g. Hepatitis B), chronic conditions (e.g. hypertension, diabetes), low weight, abnormal blood pressure, and medications that may compromise donation safety.

3. The most common behavioral reason for temporary deferral was short inter-donation intervals (Hatami et al., 2018; Kouao et al., 2012), while permanent deferrals were primarily due to risky sexual behavior (Gonçalez et al., 2013; Hatami et al., 2018; Kouao et al., 2012). Other behavioral deferral factors included drug use (Kheiri and Alibeigi, 2017), insufficient sleep, and cupping therapy (AlNouri et al., 2019).

4. For example, France’s blood transfusion system is centralized through its *Etablissement francais du sang.*

5. Supplemental Figure 2 presents an ethnographically-informed flowchart of donor recruitment, mapping the roles of hospitals, families, and DROs in Lebanon’s decentralized system.

6. French influence stems from Lebanon’s colonial history under the French Mandate (1920–1943), while American influence grew through post-independence partnerships, medical training exchanges, and aid programs in health care.

7. DROs are independent, quasi-medical NGOs that partner with hospitals on blood drives, providing logistics while hospitals supply materials, set criteria, and oversee or delegate blood collection.

8. *Houjama* (cupping therapy) is a traditional alternative practice that uses suction and small skin incisions to draw blood and promote healing.
