# Supplementary material for: Voluntary and non-remunerated blood donation by 2025? A comparative study of blood donor eligibility in 19 healthcare institutions in Lebanon
Source: J Health Psychol. 2026 Jan 23;31(10):4225–40. doi: 10.1177/13591053251408144 (PMC13451579; doi:10.1177/13591053251408144)
Supplement: Supplementary file 1 [file sj-docx-1-hpq-10.1177_13591053251408144.docx]

**Supplementary Material**

**List of figures**

[**Figure 1 -** Figure showing study context (grey-shaded areas) (2022 – 2025) vis-à-vis recent crises (black-shaded areas) in Lebanon (2018 – 2025)	2](#_Toc204645764)

[**Figure 2 -** An ethnographically informed flowchart showing the standard process of securing blood / recruiting blood donors in Lebanon.	3](#_Toc204645765)

**List of tables**

[**Table 1 -** Summary of data collected through ethnographic observations, interviews, and documents (June-September 2022).	4](#_Toc204645769)

[**Table 2 -** Data sources of study datasets (9 DHQs and 18 DSC).	4](#_Toc204645770)

[**Table 3 -** Sample characteristics.	4](#_Toc204645771)

[**Table 4 -** The four sections of the DHQs.	5](#_Toc204645772)

[**Table 5 -** Variation of information required in Section 1 of the DHQs.	5](#_Toc204645773)

[**Table 6 -** Variation of information required in Section 2 of the DHQs.	6](#_Toc204645774)

[**Table 7 -** Variation of Section 3 of the DHQs.	6](#_Toc204645775)

[**Table 8 -** Variation of information required in Section 4 of the DHQs.	7](#_Toc204645776)

[**Table 9 -** Comparison of blood donor DPRI (in days) following Covid-19 vaccination by vaccine brand and dose across 18 HIs in Lebanon.	8](#_Toc204645777)

[**Table 10 -** Table showing the minimum and maximum deferral periods for different vaccines in 18 HIs.	8](#_Toc204645778)

[**Table 11 -** List of DHQ question items on medication and treatment history.	9](#_Toc204645779)

[**Table 12 -** DPRI based on the types of medication being used for various medical conditions.	9](#_Toc204645780)

[**Table 13 -** DPRI and average intervals between successive donations among male and female donors across 18 HIs. The number of contributing HIs varied by donation type: Blood–Blood (n = 18 HIs), Blood–Platelet (n = 12 HIs), Platelet–Platelet (n = 12 HIs), Platelet–Blood (n = 11 HIs).	10](#_Toc204645781)

[**Table 14 -** Deferral period ranges associated with donors’ engagement in various lifestyle choices.	10](#_Toc204645782)

[**Table 15 -** List of DHQ question items on high-risk sexual behavior.	11](#_Toc204645783)

[**Table 16 -** Range of minimum weight requirement (Kg) across HIs by gender.	12](#_Toc204645784)

[**Table 17 -** DPRI based on travel destination.	12](#_Toc204645785)

[**Table 18 -** List of DHQ question items on travel history.	13](#_Toc204645786)

| **Contexts** | | **Years** | | | | | | | |
| --- | --- | --- | --- | --- | --- | --- | --- | --- | --- |
|  |  | **2018** | **2019** | **2020** | **2021** | **2022** | **2023** | **2024** | **2025** |
| Study context | LCBT eligibility criteria last update |  |  |  |  |  |  |  |  |
|  | National VNRD Strategy |  |  |  |  |  |  |  |  |
|  | Research fieldwork |  |  |  |  |  |  |  |  |
| Recent crises in Lebanon | Utilities crisis |  |  |  |  |  |  |  |  |
|  | October 17 revolution |  |  |  |  |  |  |  |  |
|  | Economic and financial crisis |  |  |  |  |  |  |  |  |
|  | COVID-19 pandemic |  |  |  |  |  |  |  |  |
|  | August 4 Port of Beirut Blast |  |  |  |  |  |  |  |  |
|  | Israel-Lebanon war |  |  |  |  |  |  |  |  |

Figure 1 - Figure showing study context (grey-shaded areas) (2022 – 2025) vis-à-vis recent crises (black-shaded areas) in Lebanon (2018 – 2025)

Figure 2 – An ethnographically informed flowchart showing the standard process of securing blood / recruiting blood donors in Lebanon.

Table 1 - Summary of data collected through ethnographic observations, interviews, and documents (June-September 2022).

| **Data Collected** | |
| --- | --- |
| *Ethnographic observations*  *(N=27)* | DRO spaces (call centers and common rooms) (n=19) |
|  | Blood drives (partnerships between DRO and HIs) (n=4) |
|  | DRO Events (Trainings for staff, World Blood Donor Day, etc.) (n=4) |
| *Semi-structured interviews*  *(N=30)* | Healthcare professionals (physicians, laboratory scientists) (n=6) |
|  | DRO paid staff (n=3) |
|  | DRO unpaid volunteers (n=2) |
|  | DRO administrators (directors, managers) (n=5) |
|  | Blood and plasma donors (n=7) |
|  | Non-donors (n=5) |
|  | Family members (n=2) |
| *Documents collected*  *(N=40)* | DRO Brochures (informational and educational) (n=10) |
|  | Donor Health Questionnaires (n=9) |
|  | Donor Selection Criteria (n=18) |
|  | Other documents (blood donor card, stickers, postcards) (n=3) |

Table 2 - Data sources of study datasets (9 DHQs and 18 DSC).

| **Dataset** | **Data Source (HI 1 – 19)** | | | | | | | | | | | | | | | | | | | **TOTAL** |
| --- | --- | --- | --- | --- | --- | --- | --- | --- | --- | --- | --- | --- | --- | --- | --- | --- | --- | --- | --- | --- |
|  | *1* | *2* | *3* | *4* | *5* | *6* | *7* | *8* | *9* | *10* | *11* | *12* | *13* | *14* | *15* | *16* | *17* | *18* | *19* |  |
| *DSC* | X | X | X | X | X | X | X | X | X | X | X | X | X | X | X | X | X | X |  | 18 of 19 |
| *DHQ* |  | X |  | X |  | X |  |  |  | X | X | X | X |  |  |  | X |  | X | 9 of 19 |

Table 3 – Sample characteristics.

| **Category** | **Sub-category** | **TOTAL** |
| --- | --- | --- |
| *Geographic location* | Beirut | 10 of 19 |
|  | Mount Lebanon | 5 of 19 |
|  | Kesserwan-Jbeil | 2 of 19 |
|  | South | 1 of 19 |
|  | Multiple | 1 of 19 |
| *Type of HI* | Hospital | 18 of 19 |
|  | Non-governmental organization | 1 of 19 |
| *Sector* | Public | 0 of 19 |
|  | Private | 19 of 19 |
| *Affiliations* | International | 7 of 19 |
|  | Religious | 12 of 19 |
|  | University | 13 of 19 |

Table 4 - The four sections of the DHQs.

| HI Pseudonym | Section 1: Donor information | Section 2: Physical examination assessment | Section 3: DHQ Question items | Section 4: Declaration |
| --- | --- | --- | --- | --- |
| *Control (LCBT)* | X | X | X | X |
| *HI 2* | X | X | X | X |
| *HI 4* | X | X | X | X |
| *HI 6* | X | X | X | X |
| *HI 10* |  |  | X |  |
| *HI 11* | X | X | X | X |
| *HI 12* | X | X | X | X |
| *HI 13* | X | X | X | X |
| *HI 17* | X | X | X | X |
| *HI 19* | X | X | X | X |
| TOTAL | 8 of 9 | 8 of 9 | 9 of 9 | 8 of 9 |

Table 5 - Variation of information required in Section 1 of the DHQs.

| Information Requested | HI Pseudonym | | | | | | | | | | TOTAL |
| --- | --- | --- | --- | --- | --- | --- | --- | --- | --- | --- | --- |
|  | *Control (LCBT)* | *HI2* | *HI4* | *HI6* | *HI10* | *HI11* | *HI12* | *HI13* | *HI17* | *HI19* |  |
| *First Name* | X | X | X | X | N/A | X | X | X | X | X | 8 of 9 |
| *Father’s Name* | X | X | X | X | N/A | X | X | X | X | X | 8 of 9 |
| *Family Name* | X | X | X | X | N/A | X | X | X | X | X | 8 of 9 |
| *Mother’s Name* |  |  | X |  | N/A |  |  |  | X |  | 2 of 9 |
| *Date of birth* | X | X | X | X | N/A | X | X | X |  | X | 7 of 9 |
| *Nationality/ Citizenship* | X | X | X | X | N/A | X | X | X |  | X | 7 of 9 |
| *Sex / Gender (Male/Female)* | X | X | X |  | N/A | X | X | X |  | X | 6 of 9 |
| *Home Address* | X | X | X | X | N/A | X | X | X | X | X | 8 of 9 |
| *Work Address* |  |  |  |  | N/A |  |  |  | X |  | 1 of 9 |
| *Telephone number* | X | X | X | X | N/A | X | X | X | X | X | 8 of 9 |
| *Email address* | X |  |  |  | N/A | X | X | X |  | X | 4 of 9 |
| *ID Type* | X |  |  | X | N/A | X | X | X |  | X | 5 of 9 |
| *ID Number* | X |  |  |  | N/A | X | X | X |  | X | 4 of 9 |
| *Is the donation voluntary? (Yes/No)* | X |  |  |  | N/A | X | X | X |  | X | 4 of 9 |
| *Patient’s full name* | X | X | X | X | N/A | X | X | X | X | X | 8 of 9 |
| *Relationship to patient* | X | X | X | X | N/A | X | X | X | X | X | 8 of 9 |

Table 6 - Variation of information required in Section 2 of the DHQs.

| Information Requested | HI Pseudonym | | | | | | | | | | TOTAL |
| --- | --- | --- | --- | --- | --- | --- | --- | --- | --- | --- | --- |
|  | *Control (LCBT)* | *HI 2* | *HI 4* | *HI 6* | *HI 10* | *HI 11* | *HI 12* | *HI 13* | *HI 17* | *HI 19* |  |
| *General appearance* | X | X |  | X | N/A | X | X | X |  | X | 6 of 9 |
| *Weight* | X | X | X | X | N/A | X | X | X | X | X | 8 of 9 |
| *Height* |  |  | X |  | N/A |  |  |  | X |  | 2 of 9 |
| *Temperature* | X | X | X | X | N/A | X | X | X | X | X | 8 of 9 |
| *Blood pressure (Systolic/ Diastolic)* | X | X | X | X | N/A | X | X | X | X | X | 8 of 9 |
| *Pulse* | X | X | X | X | N/A | X | X | X | X | X | 8 of 9 |
| *Needle mark on arms (Yes/No)* | X | X |  | X | N/A | X | X | X |  | X | 6 of 9 |
| *Skin lesions (Yes/No)* | X | X | X | X | N/A | X | X | X |  | X | 7 of 9 |
| *Hemoglobin / Hematocrit* | X | X | X | X | N/A | X | X | X | X | X | 8 of 9 |
| *Blood Type* |  |  | X |  | N/A |  |  |  | X |  | 2 of 9 |
| *Questionnaire review/ physical examination by (name)* | X | X |  | X | N/A | X | X | X | X | X | 7 of 9 |
| *Donor blood / components collection by (name)* |  |  |  | X | N/A |  |  |  | X |  | 2 of 9 |
| *Was the donor deferred? (Yes / No) – If yes, why?* |  |  |  | X | N/A |  |  |  | X |  | 2 of 9 |
| *Space for comments* |  | X |  | X | N/A |  |  |  |  |  | 2 of 9 |
| *Volume of blood collected* | X |  |  |  | N/A | X | X | X | X | X | 5 of 9 |

Table 7 - Variation of Section 3 of the DHQs.

| HI Pseudonym | Language(s) | | Total number of questions | Number of questions identical to LCBT DHQ | Number of revised LCBT questions | | |
| --- | --- | --- | --- | --- | --- | --- | --- |
|  | *Arabic* | *English* |  |  | *Added Questions* | *Deleted Questions* | *Modified Questions* |
| *Control (LCBT)* | X | X | 51 | N/A | N/A | N/A | N/A |
| *HI 2* | X | X | 53 | 46 | 4 | 2 | 3 |
| *HI 4* |  | X | 46 | 18 | 9 | 12 | 19 |
| *HI 6* | X | X | 21 | 12 | 5 | 33 | 4 |
| *HI 10* | X |  | 40 | 18 | 9 | 20 | 15 |
| *HI 11* | X |  | 51 | 51 | 0 | 0 | 0 |
| *HI 12* | X |  | 51 | 51 | 0 | 0 | 0 |
| *HI 13* | X |  | 51 | 51 | 0 | 0 | 0 |
| *HI 17* | X | X | 31 | 21 | 4 | 20 | 10 |
| *HI 19* | X |  | 51 | 51 | 0 | 0 | 0 |

Table 8 - Variation of information required in Section 4 of the DHQs.

| HI Pseudonym | Declaration Statement |
| --- | --- |
|  |  |
| *Control (LCBT)* | I, the undersigned, [donor name] certify that the medical history I have provided is true and accurate to the best of my knowledge. I have read and understood the educational materials on high-risk behavior and I perceive myself NOT at increased risk for the transmission of AIDS or other infectious diseases. I hereby grant permission to withdraw whole blood from me and test it as deemed necessary. If, for any reason, I feel that my blood is not safe enough to be transfused; I will notify the collecting facility. I fully understand and accept that I must remain for 10 minutes in the blood bank premises after donating whole blood. If I leave before the interval of 10 minutes, I understand it is against medical advice and any ensuing consequences are under my full responsibility. |
| *HI 2* | I, the undersigned, [donor name] certify that the medical history I have provided is true and accurate to the best of my knowledge. I have read and understood the educational materials on high-risk behavior and I perceive myself NOT at increased risk for the transmission of AIDS or other infectious diseases. I hereby grant permission to withdraw whole blood from me and test it as deemed necessary. If, for any reason, I feel that my blood is not safe enough to be transfused; I will notify the collecting facility. I fully understand and accept that I must remain for 10 minutes in the blood bank premises after donating whole blood. If I leave before the interval of 10 minutes, I understand it is against medical advice and any ensuing consequences are under my full responsibility. |
| *HI 4* | I, the undersigned, [donor name], certify that the information I have provided is correct, accurate, and complete to the best of my knowledge. I understand that answering the above questions clearly and honestly contributes to my safety and to that of the person whom I am donating blood. |
| *HI 6* | I, the undersigned, declare that the above information is true and I take full responsibility in case it proved otherwise. I understand that blood donation is a totally voluntary act and that no inducement or remuneration has been offered. Donation of blood components is a medical procedure and by donating voluntarily, I accept the risks associated with it. I have also understood that the donated blood will be the property of the healthcare institution and that in case the blood was not given to the designated patient, I will not have the right to take back any blood units. |
| *HI 10* | N/A |
| *H 11* | I, the undersigned, [donor name] certify that the medical history I have provided is true and accurate to the best of my knowledge. I have read and understood the educational materials on high-risk behavior and I perceive myself NOT at increased risk for the transmission of AIDS or other infectious diseases. I hereby grant permission to withdraw whole blood from me and test it as deemed necessary. If, for any reason, I feel that my blood is not safe enough to be transfused; I will notify the collecting facility. I fully understand and accept that I must remain for 10 minutes in the blood bank premises after donating whole blood. If I leave before the interval of 10 minutes, I understand it is against medical advice and any ensuing consequences are under my full responsibility. |
| *H 12* | I, the undersigned, [donor name] certify that the medical history I have provided is true and accurate to the best of my knowledge. I have read and understood the educational materials on high-risk behavior and I perceive myself NOT at increased risk for the transmission of AIDS or other infectious diseases. I hereby grant permission to withdraw whole blood from me and test it as deemed necessary. If, for any reason, I feel that my blood is not safe enough to be transfused; I will notify the collecting facility. I fully understand and accept that I must remain for 10 minutes in the blood bank premises after donating whole blood. If I leave before the interval of 10 minutes, I understand it is against medical advice and any ensuing consequences are under my full responsibility. |
| *H 13* | I, the undersigned, [donor name] certify that the medical history I have provided is true and accurate to the best of my knowledge. I have read and understood the educational materials on high-risk behavior and I perceive myself NOT at increased risk for the transmission of AIDS or other infectious diseases. I hereby grant permission to withdraw whole blood from me and test it as deemed necessary. If, for any reason, I feel that my blood is not safe enough to be transfused; I will notify the collecting facility. I fully understand and accept that I must remain for 10 minutes in the blood bank premises after donating whole blood. If I leave before the interval of 10 minutes, I understand it is against medical advice and any ensuing consequences are under my full responsibility. |
| *HI 17* | I declare on my responsibility that the information I have provided is correct and I grant permission to the collecting facility to withdraw whole blood from me and test it as deemed necessary. |
| *HI 19* | I, the undersigned, [donor name] certify that the medical history I have provided is true and accurate to the best of my knowledge. I have read and understood the educational materials on high-risk behavior and I perceive myself NOT at increased risk for the transmission of AIDS or other infectious diseases. I hereby grant permission to withdraw whole blood from me and test it as deemed necessary. If, for any reason, I feel that my blood is not safe enough to be transfused; I will notify the collecting facility. I fully understand and accept that I must remain for 10 minutes in the blood bank premises after donating whole blood. If I leave before the interval of 10 minutes, I understand it is against medical advice and any ensuing consequences are under my full responsibility. |

Table 9 - Comparison of blood donor DPRI (in days) following Covid-19 vaccination by vaccine brand and dose across 18 HIs in Lebanon.

| **Covid-19 vaccine brand** | **DPRI after specific dose (in days)** | | |
| --- | --- | --- | --- |
|  | **Dose 1** | **Dose 2** | **Dose 3** |
| *AstraZeneca* | 2 – 60 | 0 – 60 | 0 – 30 |
| *Johnson & Johnson* | 2 – 60 | 0 – 60 | 0 – 30 |
| *Moderna* | 2 – 60 | 0 – 60 | 0 – 30 |
| *Pfizer* | 0 – 60 | ½ – 60 | 0 – 30 |
| *Sinopharm* | 2 – 60 | 0 – 60 | 0 – 30 |
| *Sputnik V* | 7 – 60 | ½ – 60 | 0 – 30 |

Table 10 - Table showing the minimum and maximum deferral periods for different vaccines in 18 HIs.

| **Other vaccines** | **Control (LCBT)** | **HI DPRI** |
| --- | --- | --- |
| *Human Papillomavirus* | ID | ID – 3 months |
| *Influenza* | 28 days | 5 days – 3 months |
| *Diphtheria, Tetanus, Pertussis (DPT/Tdap)* | ID | 1 month – 12 months |

Table 11 - List of DHQ question items on medication and treatment history.

| Source | Question number | Questions on sexual behavior |
| --- | --- | --- |
| *TRANSPOSE* | 2.9 | Are you using or have you used any prescribed or over the counter medication in the last 3 months (e.g. birth control, pain killers)? |
|  | 2.12 | Prior to 1986, have you been treated with hormones of the pituitary gland, e.g., because of growth disturbance, infertility, endometriosis? |
|  | 2.13 | Have you ever been diagnosed or treated for anaemia or iron deficiency? |
| *LCBT*  *(H11, 12, 13, and 19)* | 2 | Are you currently taking antibiotics or any other medication for an infection? |
|  | 3 | Are you currently taking or have you ever taken any medication for any reason? If yes list |
|  | 27 | In the past 12 months, have you been given Hepatitis B Immune Globulin (HBIG)? |
|  | 28 | In the past 12 months, did you have or had you been treated for Syphilis or Gonorrhea? |
|  | 39 | In the past 12 months, have you used clotting factor concentrates for bleeding problems? |
|  | 41 | In the past 12 months, have you received Growth Hormone? |
| *HI 2* | 2 | Are you currently taking antibiotics or any other medication for an infection? |
|  | 3 | Are you currently taking or have you ever taken any medication for any reason? If yes list |
|  | 25 | In the past 12 months, have you been given Hepatitis B Immune Globulin (HBIG)? |
|  | 26 | In the past 12 months, did you have or had you been treated for Syphilis or Gonorrhea? |
|  | 39 | Have you ever used clotting factor concentrates for bleeding problems? |
|  | 41 | Have you ever received Growth Hormone (made from human origin)? |
| *HI 4* | 4 | In the past two weeks have you taken any antibiotics, antihistamines, or ant-inflammatory medication? |
|  | 5 | Are you on any other chronic medication? |
|  | 29 | In the past year, have you been treated for Syphilis or Gonorrhea? |
|  | 41 | Have you ever used clotting factor concentrates for bleeding problems? |
|  | 44 | Have you ever had diabetes and take insulin treatment? |
| *HI 6* | 4 | Are you currently taking antibiotics or any other medication for an infection? |
|  | 5 | Are you currently taking or have taken any medication for any reason? If yes list |
|  | 21 | In the past 12 months, have you been given Hepatitis B Immune Globulin (HBIG)? |
| *HI 10* | 2 | Are you currently taking antibiotics or any other medication for an infection? |
|  | 3 | Are you currently taking or have taken any medication for any reason? If yes list |
|  | 4 | Have you taken any medications listed on the list of medications that lead to postponement of the donation process during the periods specified on the list? |
|  | 6 | In the past 48 hours, did you take Aspirin or any other medication that contains Aspirin? |
| *HI 17* | 3 | Are you currently taking antibiotics or any other medication for an infection? |
|  | 17 | Have you ever taken antibiotics against Hepatitis A Virus? |
|  | 18 | Have you ever been treated for Syphilis or other sexual diseases? |

Table 12 – DPRI based on the types of medication being used for various medical conditions.

| **Medical condition / Medication used** | **Control (LCBT)** | **DPRI** |
| --- | --- | --- |
| *Acne* | ID | 1 month – 3 years |
| *ADHD* | N/A | ID – PD |
| *Allergy* | ID – PD | ID – PD |
| *Asthma* | ID – 14 days | ID – PD |
| *Bipolar disorder* | N/A | PD |
| *Coronary artery disease / Cholesterol* | PD | ID – PD |
| *Depression* | ID | ID – PD |
| *Diabetes* | ID – PD | ID – PD |
| *Epilepsy* | ID | ID – PD |
| *Hormones* | PD | 1 month – PD |
| *Hypertension* | ID – PD | ID – PD |
| *Migraine* | N/A | 2 days – PD |
| *Previous infection with Covid-19 (following negative test result for PCR)* | N/A | 2 days – 2 months |
| *Schizophrenia* | N/A | PD |
| *Thyroid* | ID – PD | ID – PD |
| *Triglycerides* | N/A | ID – PD |

Table 13 - DPRI and average intervals between successive donations among male and female donors across 18 HIs. The number of contributing HIs varied by donation type: Blood–Blood (n = 18 HIs), Blood–Platelet (n = 12 HIs), Platelet–Platelet (n = 12 HIs), Platelet–Blood (n = 11 HIs).

|  | | **Males** | | **Females** | |
| --- | --- | --- | --- | --- | --- |
| **Successive donations** | **Control (LCBT)** | **DPRI** | ***Average*** | **DPRI** | ***Average*** |
| *Blood and blood* | 2 months | 2 months – 3 months | *2.3 months* | 2 months – 4 months | *2.9 months* |
| *Blood and platelet* | 2 months | 10 days – 2 months | *1.6 months* | 10 days – 3 months | *1.7 months* |
| *Platelet and platelet* | 2 days – 2 months * | 3 days – 1 month | *14.5 days* | 3 days – 1 month | *14.5 days* |
| *Platelet and blood* | 2 days – 2 months * | 2 days – 2 months | *17 days* | 2 days – 2 months | *17.2 days* |

* After a platelet donation, a minimum interval of 48 hours is required before any further donation if red blood cells were successfully returned during the apheresis. If red cell return was incomplete, an 8-week (2 months) deferral is required, unless the total red cell loss was less than 200 mL.

Table 14 - Deferral period ranges associated with donors’ engagement in various lifestyle choices.

| **Lifestyle** | **DPRI** | |
| --- | --- | --- |
|  | **Control (LCBT)** | **HIs** |
| *Laser for hair removal* | No restrictions | ID – PD |
| *Electrical epilation for hair removal* | No restrictions | ID – 6 months |
| *Protein shakes* | No restrictions | ID – PD |
| *Fat burner* | No restrictions | ID – PD |
| *Alcohol* | ID – 12 hours | 12 hours – 48 hours |
| *Weight loss medication* | No restrictions | 2 weeks – 4 weeks |
| *Houjama (cupping therapy)* | 1 year | 2 months – 12 months |
| *Acupuncture* | 1 year | 1 year |
| *Tattoo* | 1 year | 3 months – 12 months |
| *Piercing* | 1 year | 3 months – 12 months |

Table 15 - List of DHQ question items on high-risk sexual behavior.

| Source | Question number | Questions on sexual behavior |
| --- | --- | --- |
| *TRANSPOSE* | 6.5 | Have you in the last 6 months been paid or been given payment, for example with money or drugs, for sex? |
|  | 6.6 | Did you in the last 6 months have sex with someone? |
|  | 7.1 | [Male donors only]: Have you had sex with another man in the past 6 months? |
|  | 8.1 | [Female donors only]: Have you in the last 6 months had sex with a man, who to your knowledge, has previously had sex with another man? |
| *Control (LCBT)*  *And H1s 11, 12, 13, and 19* | 20 | In the past 12 months, have you had sexual contact with anyone who has HIV/AIDS or has had a positive test for the HIV/AIDS virus? |
|  | 21 | In the past 12 months, have you had sexual contact with a prostitute or anyone else who takes money or drugs or other payment for sex? |
|  | 22 | In the past 12 months, have you had sexual contact with anyone who has ever used needles to take drugs, steroids, or anything NOT prescribed by a doctor? |
|  | 23 | In the past 12 months, have you had sexual contact with anyone who has hemophilia or has taken clotting factor concentrates for bleeding problems? |
|  | 24 | [Female donors only]: In the past 12 months, have you had sexual contact with a male who has ever had sexual contact with another male? |
|  | 25 | In the past 12 months, have you had sexual contact with a person who has Hepatitis or yellow jaundice? |
|  | 33 | From 1977 to the present, have you, received money, drugs or other payment for sex? |
|  | 34 | [Male donors only]: From 1977 to the present, have you, had sexual contact with another male, even once? |
| *HI 2* | 18 | In the past 12 months have you had sexual contact with anyone who has HIV/AIDS or has had a positive test for HIV/AIDS virus? |
|  | 19 | In the past 12 months have you had sexual contact with prostitute or anyone else who takes money or drugs or other payment for sex? |
|  | 20 | In the past 12 months have you had sexual contact with anyone who has ever used needles to take drugs, steroids, or anything NOT prescribed by a doctor? |
|  | 21 | In the past 12 months have you had sexual contact with anyone who has hemophilia or has taken clotting factor concentrates for bleeding problems? |
|  | 22 | [Female donors only]: In the past 12 months have you had sexual contact with anyone who has ever had sexual contact with another male? |
|  | 23 | In the past 12 months have you had sexual contact with a person who has Hepatitis or yellow jaundice? |
|  | 32 | From 1977 to the present, have you, received money, drugs or other payment for sex? |
|  | 35 | Have you ever had sexual contact with anyone who was born or lived in Africa? |
| *HI 4* | 23 | In the past year, have you had sexual contact with anyone who has HIV/AIDS or has had a positive test for HIV/AIDS virus? |
|  | 24 | In the past year, have you had sexual contact in exchange for money, drugs, or other payment? |
|  | 25 | In the past year, have you had sexual contact with anyone who has ever used needles to take drugs, steroids, or anything not prescribed by a doctor? |
|  | 26 | [Male donors only]: In the past year, have you had sexual contact with another male? |
|  | 27 | [Female donors only]: In the past year, have you had sexual contact with a male who had sexual contact with another male? |
|  | 28 | In the past year, have you been in contact with someone who had hepatitis? (sexual contact, family members, neighbors…?) |
| *HI 6* | 18 | In the past 12 months, have you had sexual contact with a prostitute or anyone else who takes money or drugs or other payments for sex? |
|  | 22 | In the last 3 years, have you been to Africa or had a sexual relation with someone who lived in Africa? |
| *HI 10* | 15 | In the past 3 months, have you had sexual contact with anyone who has HIV/AIDS or has had a positive test for HIV/AIDS virus? |
|  | 16 | In the past 3 months, have you had sexual contact with prostitute or anyone else who takes money or drugs or other payment for sex? |
|  | 17 | In the past 3 months, have you had sexual contact with anyone who has ever used needles to take drugs, steroids, or anything NOT prescribed by a doctor? |
|  | 18 | [Male donors only]: In the past 3 months, have you had sexual contact with another male? |
|  | 19 | [Female donors only]: In the past 3 months, have you had sexual contact with a male who had sexual contact with another male? |
|  | 24 | In the past 3 months, have you, received money, drugs or other payment for sex? |
|  | 26 | In the past 12 months, have you had sexual contact with a person who has Hepatitis or yellow jaundice? |
| *HI 17* | 15 | Have you ever had sexual contact with anyone who has HIV/AIDS? Or receives money for sex? |
|  | 16 | Have you ever lived with or had sexual contact with anyone who has Hepatitis or yellow jaundice? |
|  | 21 | Have you ever had sex with a person who has lived in Africa? |

Table 16 - Range of minimum weight requirement (Kg) across HIs by gender.

|  | | **Range interval of minimum donation weight (Kg)** |
| --- | --- | --- |
| *Control (LCBT)* | | 50 (without any reported gender differences) |
| *HIs* | Males | 50 – 65 |
|  | Females | 50 – 70 |

Table 17 - DPRI based on travel destination.

| **Geographic region/ country listed by HIs** | **LCBT risk assessment** | | | | | | **HI DPRI** |
| --- | --- | --- | --- | --- | --- | --- | --- |
|  | *Disease* | Malaria | WNV | Chikungunya | Chagas | Dengue |  |
|  | *DPRI* | 1 year – PD | 6 months | 28 days – 6 months | PD | 28 days – 6 months |  |
| *Algeria* |  |  |  |  |  |  | 1 year |
| *Brazil* |  | X |  |  | X |  | 1 year – PD |
| *China* |  | X |  |  |  |  | 1 week – 1 year |
| *Egypt* |  |  |  |  |  |  | ID – PD |
| *“Europe”* |  |  |  |  |  |  | ID – PD |
| *Hungary* |  |  |  |  | X |  | ID – PD |
| *India* |  | X |  | X |  |  | ID – 1 year |
| *Indonesia* |  | X |  |  |  |  | 1 year |
| *Iraq* |  | X |  |  |  |  | ID – PD |
| *Malaysia* |  | X |  | X |  |  | 1 year |
| *Mexico* |  | X |  |  | X |  | 1 year |
| *Nigeria* |  | X |  |  |  |  | ID – PD |
| *Pakistan* |  | X |  |  |  |  | 3 years |
| *Philippines* |  | X |  |  |  |  | ID – PD |
| *South Africa* |  | X |  |  |  |  | ID – PD |
| *Sri Lanka* |  | X |  | X |  |  | 1 year |
| *Syria* |  |  |  |  |  |  | ID – 1 week |
| *Thailand* |  | X |  |  |  |  | 1 week – 1 year |
| *Vietnam* |  | X |  |  |  |  | 1 year |
| *Yemen* |  | X |  |  |  |  | ID |

Table 18 - List of DHQ question items on travel history.

| Source | Question number | Questions on travel history |
| --- | --- | --- |
| *TRANSPOSE* | 4.1 | Have you lived outside this country for more than 6 months continuously (for example if you were born in another country)? |
|  | 5.1 | Have you traveled outside this country during the last 12 months? Were you well there and after your return? |
|  | 5.2 | Have you stayed in the UK for more than 6 months in total between 1 January 1980 and 31 December 1996? |
| *LCBT*  *(H11, 12, 13, and 19)* | 30 | In the past 3 years, have you been outside Lebanon? If yes, where? |
|  | 31 | From 1980 through 1996, did you spend time that adds up to 3 months or more in the United Kingdom? |
|  | 32 | From 1980 to the present, have you received a blood transfusion in Europe? |
|  | 35 | Have you ever been to or lived in any endemic country for Malaria? |
| *HI 2* | 28 | In the past 3 years, have you been outside Lebanon? |
|  | 29 | From 1980 through 1996, did you spend time that adds up to 3 months or more in the United Kingdom? |
|  | 30 | From 1980 to the present, did you spend time that adds up to 5 years or more in Europe? |
|  | 31 | From 1980 to the present, did you receive a blood transfusion in Europe? |
|  | 34 | Have you ever been to or lived in any African country? |
|  | 35 | Have you ever had sexual contact with anyone who was born or lived in Africa? |
| *HI 4* | 31 | Between 1980 and 1996, did you spend time that adds up to 3 months or more in the United Kingdom? |
|  | 32 | From 1980 until the present, have you spent time that exceeds 5 years in Europe? |
|  | 33 | From 1980 until the present, have you ever received a blood transfusion in the UK or France? |
|  | 38 | From 1977 to the present, have you been or lived in any African country? |
| *HI 6* | 22 | In the last 3 years, have you been to Africa or had a sexual contact with someone who lived in Africa? |
| *HI 10* | 29 | In the past 3 years, have you traveled to: Europe? Africa? Australia? North America? South America? Asia? |
|  | 30 | Between 1980 and 1996, did you spend time that adds up to 3 months or more in the United Kingdom? |
|  | 31 | Between 1980 and 2001, have you spent time that exceeds 5 years in France or Ireland? |
|  | 32 | From 1980 until the present, have you ever received a blood transfusion in the UK or France? |
| *HI 17* | 19 | In the past 3 years, have you been outside Lebanon? |
|  | 20 | In the past 3 years, did you travel to or live in Africa? |
|  | 21 | In the past 3 years, did you have sexual contact with anyone who was born or lived in Africa? |
